# Subnanometer structure of medusavirus capsid during maturation using cryo-electron microscopy

**DOI:** 10.1128/jvi.00436-24

**Published:** 2024-08-28

**Authors:** Ryoto Watanabe, Chihong Song, Masaharu Takemura, Kazuyoshi Murata

**Affiliations:** 1 Department of Physiological Sciences, School of Life Science, The Graduate University for Advanced Studies (SOKENDAI), Okazaki, Aichi, Japan; 2 Exploratory Research Center on Life and Living Systems (ExCELLS), National Institutes of Natural Sciences, Okazaki, Aichi, Japan; 3 National Institute for Physiological Sciences, National Institutes of Natural Sciences, Okazaki, Aichi, Japan; 4 Institute of Arts and Sciences, Tokyo University of Science, Shinjuku, Tokyo, Japan; Michigan State University, East Lansing, Michigan, USA

**Keywords:** cryo-electron microscopy, giant virus, single particle analysis, block-based reconstruction, virus capsid, major capsid protein, minor capsid proteins, penton protein, spikes

## Abstract

Medusavirus is a giant virus classified into an independent family of *Mamonoviridae*. Amoebae infected with medusavirus release immature particles in addition to virions. These particles were suggested to exhibit the maturation process of this virus, but the structure of these capsids during maturation remains unknown. Here, we apply a block-based reconstruction method in cryo-electron microscopy (cryo-EM) single particle analysis to these viral capsids, extending the resolution to 7–10 Å. The maps reveal a novel network composed of minor capsid proteins (mCPs) supporting major capsid proteins (MCPs). A predicted molecular model of the MCP fitted into the cryo-EM maps clarified the boundaries between the MCP and the underlining mCPs, as well as between the MCP and the outer spikes, and identified molecular interactions between the MCP and these components. Several structural changes of the mCPs under the fivefold vertices of the immature particles were observed, depending on the presence or absence of the underlying internal membrane. In addition, the lower part of the penton proteins on the fivefold vertices was also missing in mature virions. These dynamic conformational changes of mCPs indicate an important function in the maturation process of medusavirus.

**IMPORTANCE:**

The structural changes of giant virus capsids during maturation have not thus far been well clarified. Medusavirus is a unique giant virus in which infected amoebae release immature particles in addition to mature virus particles. In this study, we used cryo-electron microscopy to investigate immature and mature medusavirus particles and elucidate the structural changes of the viral capsid during the maturation process. In DNA-empty particles, the conformation of the minor capsid proteins changed dynamically depending on the presence or absence of the underlying internal membranes. In DNA-full particles, the lower part of the penton proteins was lost. This is the first report of structural changes of the viral capsid during the maturation process of giant viruses.

## INTRODUCTION

Medusaviruses belong to the phylum *Nucleocytoviricota* and were first discovered in hot spring water in Japan (1). Uniquely, the genome of medusaviruses contains a complete set of histone proteins, encoding four core histones and one linker histone, and a DNA polymerase that is located at the root of the eukaryotic clade (1). This fact suggests that medusaviruses are phylogenetically closer to eukaryotes than other giant viruses on the phylum *Nucleocytoviricota*. Therefore, medusaviruses were initially classified as an independent family, *Medusaviridae,* and later reclassified as the family *Mamonoviridae* in the phylum *Nucleocytoviricota* (2). *Medusavirus stheno*, a sister strain of medusavirus, was subsequently isolated from a river in Kyoto, Japan (3), indicating that medusaviruses are more widely distributed throughout a range of environments.

Medusaviruses also show unique features in their replication and particle structure. In our previous study, we identified four types of medusavirus particles (pseudo-empty, empty, semi-full, and full DNA particles) in the culture medium of medusavirus-infected amoebae (4). Time-lapse observation of medusavirus-infected amoeba cells using conventional transmission electron microscopy demonstrated that these four types of particles illustrate the maturation process of virus particles. At the early stage of infected amoeba cells, pseudo-DNA-empty particles appear in the cytoplasm, and the substance in the particles is released to form DNA-empty particles. They then begin to be filled with viral DNA (semi-DNA-full particles). Eventually, the capsid is completely full of viral DNA (DNA-full particle), and the mature DNA-full particles are released outside the cell by exocytosis together with immature particles (pseudo-DNA-empty, DNA-empty, and semi-DNA-full particles) (1, 4). These medusavirus particles commonly display a T = 277 icosahedral capsid with a diameter of approximately 260 nm, surrounded by spikes with lengths of approximately 14 nm (1, 4). Single particle analysis (SPA) by cryo-electron microscopy (cryo-EM) has resolved the structures of the DNA-full and DNA-empty particles at a resolution of 19.5 Å and 21.5 Å, respectively. Although the DNA-full particles were approximately 1 nm smaller than the DNA-empty particles, these particles appear structurally similar to capsids at these resolutions.

Capsid structures of icosahedral giant viruses have also been studied by cryo-EM SPA. Icosahedral capsids are commonly composed of a combination of 12 pentasymmetrons and 20 trisymmetrons (5). For the capsid formation of icosahedral giant viruses, a spiral assembly pathway has been proposed in which particle assembly is initiated in a spiraling fashion around the fivefold vertices, based on the structural orientation of the major capsid protein (MCP) capsomers on the pentasymmetron (6). *Paramecium Bursaria* Chlorella Virus 1 (PBCV-1), African swine fever virus (ASFV), and Singapore grouper iridovirus (SGIV) have been successfully reported at near-atomic resolution using cryo-EM SPA, and not only the MCPs but also the minor capsid proteins (mCPs) that support the MCPs have been identified (7–9). In these reports, a “block-based” reconstruction method was commonly used (10), which can divide large objects of high symmetry with large defocus gradients into several smaller blocks based on the symmetry axes of the icosahedral capsid and reconstructed them independently in three dimensions (3D). As a result, the resolution limit imposed by the Ewald sphere curvature effect can be extended. Higher resolution maps revealed that mCPs form a complex molecular network under the MCP layer, directly supporting the MCP array and supporting viral DNA through the internal membrane (IM). In PBCV-1, mCPs named P3, P4, and P5 form a trapezoidal unit, which further forms a large trisymmetron. In giant viruses of *Marseilleviridae*, the trapezoidal units were connected through another mCP named “cement component” to form a trisymmetron (11, 12). In contrast, the trisymmetron of ASFV was constructed of an mCP network consisting of a single mCP (p17) (13).

In this study, we used cryo-EM SPA and block-based reconstruction method to present the capsid structure of medusavirus at 7.3–9.9 Å resolution. The cryo-EM maps revealed the boundaries between the MCP and the inner mCP components, and between the MCP and the outer spikes. Potentially interacting loops of these connections were identified in the MCP. We also identified a medusavirus-specific mCP network that is distinct from known mCP networks of other giant viruses. Under the fivefold axis, we further observed structural changes in the pentasymmetron mCP components in the presence or absence of the IM, and a portion of the penton protein was lost in DNA-full particles when compared with DNA-empty particles. These results suggest structural changes of the capsid proteins during the virus particle formation and propose a new model of the maturation process of giant viruses.

## RESULTS

### Subnanometer resolution structures of DNA-empty and DNA-full particles

**Fig 1:**
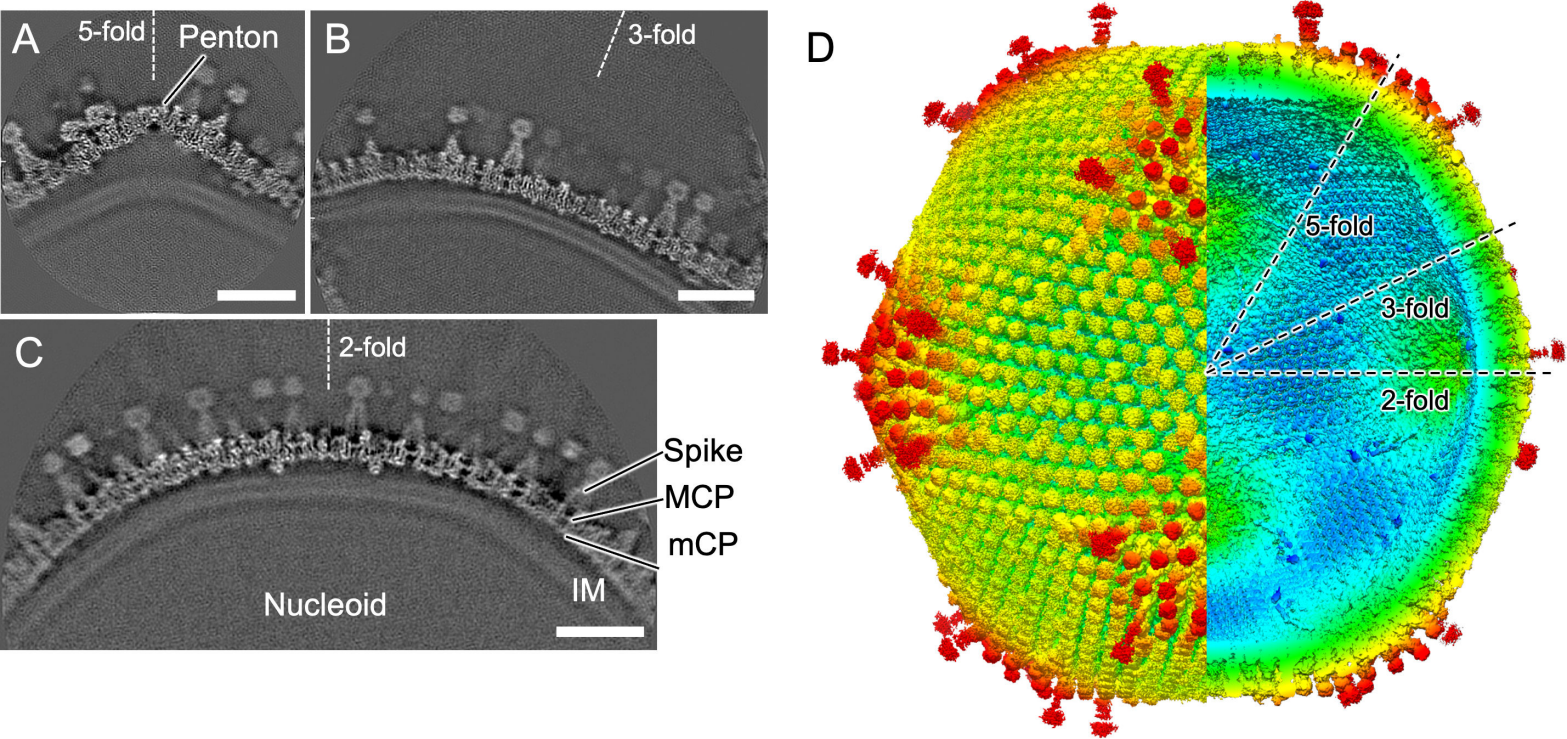
Subnanometer resolution structure of medusavirus capsid. (A, B, and C) Each slice of the block-based reconstruction map is centered on the fivefold, threefold, and twofold axes. Under the capsid, the internal membrane (IM) encases the nucleoid. The capsid consists of the MCPs, the spikes, the mCPs, and the penton proteins. Scale bars = 20 nm. (D) The whole medusavirus capsid merged with each capsid map is generated by block-based reconstruction individually. Maps are colored by radius. The icosahedral fivefold, threefold, and twofold axes are indicated by dotted lines.

In our previous study, we used 4,551 DNA-empty and 6,981 DNA-full particles to reconstruct the structure of medusavirus particles at a resolution of 21.5 Å and 19.5 Å, respectively, by imposing icosahedral symmetry (4). In this study, we applied a block-based reconstruction method to these cryo-EM data, extracting images around the fivefold, threefold , and twofold symmetry axes of the icosahedral particles separately and reconstructing them in 3D. The resolution previously limited by the defocus gradient was extended to 7.3–9.9 Å (Table 1; Fig. S1; Fig. 1A through C). By combining each block, a complete picture of the medusavirus particle was constructed (Fig. 1D). In addition to DNA-full and DNA-empty particles, the maps were classified by structures with and without an IM (Fig. S1C and D).

**TABLE 1:** Cryo-EM data set

Parameter	Description or value	
Microscope	Titan Krios G2	
Accelerating voltage (kV)	300	
Spherical aberration (mm)	0.1 (Cs corrector)	
Detector	Falcon III	
Total dose (e^−^/A^2^)	30	
Micrographs	2,084	
Frames per micrograph	40	
Nominal magnification	22,500×	
Pixel spacing on the specimen (Å/pixel)	3.03	

	DNA-empty particle	DNA-full particle
Initial particles picked	4,625	7,038
Final particles used	4,551	6,981
Symmetry imposed	I1	I1
Resolution (Å)	21.5	19.5

### Network structure of the minor capsid proteins

**Fig 2:**
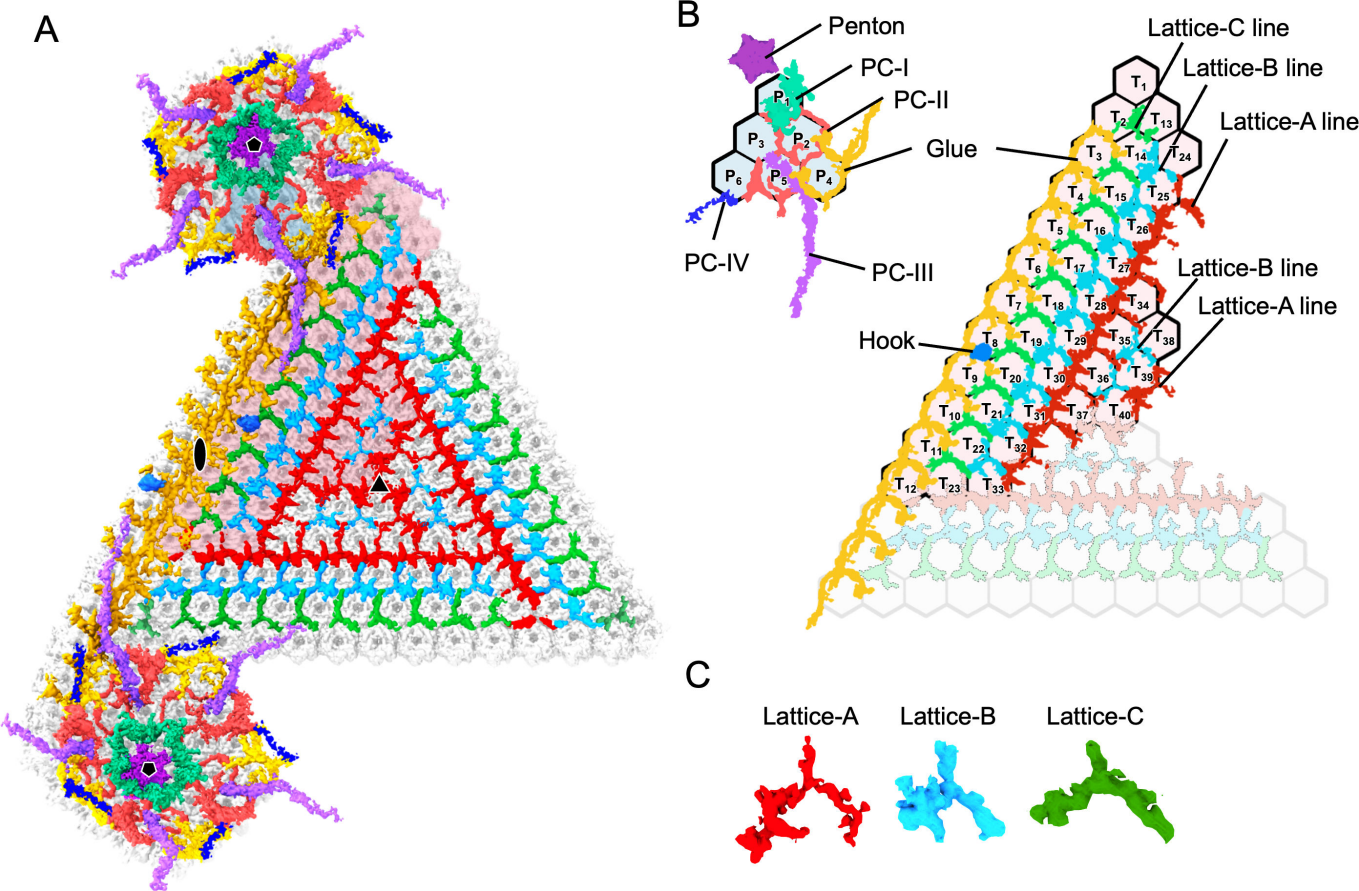
Minor capsid protein network of medusavirus capsid. (**A**) Cryo-EM map of mCPs and penton proteins as viewed from inside the pentasymmetron and trisymmetron. Fivefold, threefold, and twofold axes are shown as pentagon, triangle, and ellipses, respectively. (**B**) Schematic diagram of mCPs and penton proteins as viewed from inside the capsid. Each mCP and penton protein is classified into components based on their feature and/or function and color-coded as follows: penton (purple), PC-I (turquoise), PC-II (salmon), PC-III (orchid), PC-IV (blue), Lattice-A (red), Lattice-B (light sky blue), Lattice-C (green), Glue (yellow), and Hook component (royal blue). The hexagonal lattices show the location of MCP trimers. Individual trimers are labeled P1 to P6 in the asymmetric unit of pentasymmetron and T1 to T40 in the asymmetric unit of trisymmetron. (**C**) Three lattice components, Lattice-A, Lattice-B, and Lattice-C, which mainly form the mCP network in the trisymmetron under the MCPs, are extracted.

The mCPs form a unique protein network beneath the MCP trimer arrays of the pentasymmetron and trisymmetron, respectively (Fig. 2A). Although proteomic analysis of the medusavirus virion revealed 80 virion proteins (1), their characteristics, such as copy number and hydrophobicity, have not been determined. Furthermore, the current resolution map does not allow for the identification of individual proteins. Therefore, here, we arbitrarily segmented these protein complexes based on their structural features and predicted functions and named them as components. In the pentasymmetron, the penton proteins, which are located at the center of the fivefold vertices, are easily identified (purple in Fig. 2A and B). The peripheral mCP complexes were divided into four pentasymmetron components, named PC-I, PC-II, PC-III, and PC-IV, based on their location and function (Fig. 2B). PC-Is are located closest to the penton and support an array of MCP trimers, labeled P1 (turquoise green in Fig. 2B). PC-IIs are located around PC-Is and support the array of other MCP trimers, labeled P3 to P5 (salmon pink in Fig. 2B). PC-IIIs and PC-IVs are filamentous components located further below the mCP network of PC-Is and PC-IIs, connecting the pentasymmetron to the trisymmetron and the IMs, which will be discussed in a later section (orchid and blue in Fig. 2).

In the trisymmetron, the mCP network was formed by a combination of three slightly different lattice components, named Lattice-A, Lattice-B, and Lattice-C, respectively (red, light blue, and green in Fig. 2C). These are repeatedly connected in series around the threefold axis in the order of Lattice-A, Lattice-B, Lattice-A, and Lattice-B lines from the center to the periphery (Fig. 2B). In addition, lanes of Lattice-C components surrounded this repeat line of Lattice-A and Lattice-B (Fig. 2B). The Lattice-C lines near the corners of the trisymmetron were deformed due to the interference of the components of the pentasymmetron (Fig. S2). This indicates a unique mCP network in trisymmetron of the giant virus, where the repeat lattice lines form a large trisymmetron. The lattice lines of the Glue components (yellow in Fig. 2) are connected at the interfaces of the pentasymmetron and the trisymmetron. Although these four lattice-forming components are functionally distinct, they are structurally similar and appear to be composed of similar protein complexes that adopt different conformations. Hook component in the middle of the lattice line of the Glue component connects the viral capsid and the IM (sky blue in Fig. 2). More quantitative approaches involving computational modeling and statistical shape analysis are further required to identify individual mCPs within each component.

### MCP structure of medusavirus

**Fig 3:**
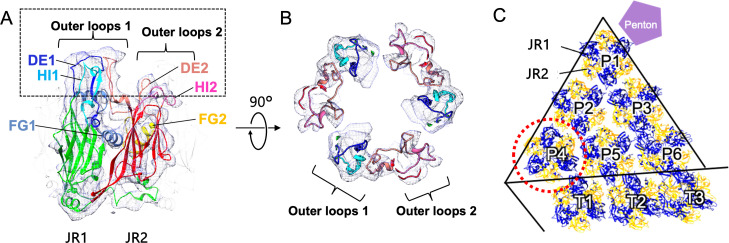
Structure of medusavirus MCP. (**A**) Cryo-EM map of the MCP monomer and fitting of the predicted model. The outer loops of the model are color-coded as follows: DE1 (blue), FG1 (light sky blue), HI1 (cyan), DE2 (salmon), FG2 (yellow), and HI2 (hot pink). The outer loops 1 were composed of DE1, HI1 loops, and the first half of DE2 on JR1, and the outer loops 2 were composed of the second half of DE2 and HI2 loops on JR2, respectively. The unique third α-helix in FG1 loop of medusavirus MCP was located between JR1 and JR2. (**B**) Top view of the MCP trimer in the dashed box region in A. (**C**) The MCP labeled P4 (dotted red circle) is rotated by 60° relative to other MCPs in the asymmetric unit of the pentasymmetron.

**Fig 4:**
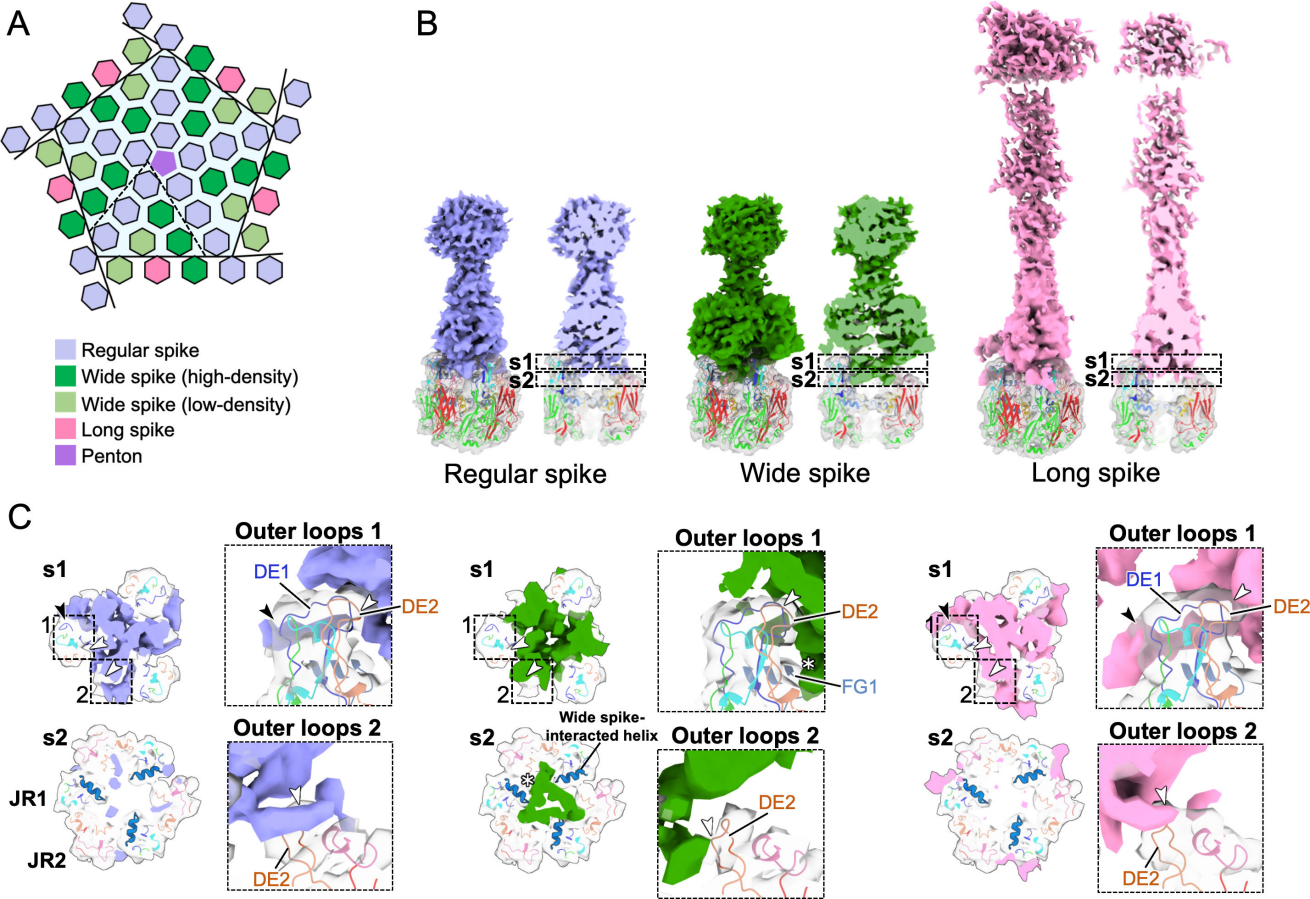
Three types of spikes around fivefold axis and their interactions with MCPs. (**A**) Schematic diagram of MCP trimers (hexagons) arranged around the penton (pentagon) at the fivefold axis. Each MCP trimer is shown in a different color according to the spike type (**B**). (**B**) Structures of three types of spikes on the MCP trimer (bottom). Shown are regular spike (lilac), wide spike (green), and long spike (pink). (**C**) Sliced views of each type of spikes, cutoff by dashed boxes s1 and s2 in panel B, and focused views of the dashed boxes 1 and 2. Showing interactions of each spike with outer loops 1 and 2, and the third FG1 α-helix of each MCP.

The amino acid sequence of medusavirus MCP showed high homology to that of PBCV-1. Furthermore, pairwise alignment with the PBCV-1 MCP predicted that the MCP has the same "double jelly roll" motif composed of jelly roll 1 (JR1) and jelly roll 2 (JR2) (Fig. 3; Fig. S3). A molecular model of MCP was generated by AlphaFold2 (14), and the resulting model was fitted into the cryo-EM map and modified based on the MCP density using COOT (15, 16) and Phenix (17). Portions of the generated MCP model that was extended or unoccupied for the cryo-EM map were manually modified in COOT, and the feasibility of the structure was evaluated and energetically optimized in Phenix. As a result, the medusavirus MCP model was fitted to the cryo-EM map with the outer loops 1 consisting of DE-1, the first half of DE-2, and HI-1 on JR1 (Fig. 3A and B). In addition, outer loops 2 were composed of the second half of DE-2 and HI-2 on JR2. These loops form large density clusters on the MCP trimer. As described below, these clusters of the outer loops (Fig. 3A and B) and the unique third α-helix in the FG-1 loop (Fig. 3; Fig. S3) showed different interactions with different types of spikes (Fig. 4). Further, the MCP labeled P4 was confirmed to be rotated by 60° relative to other MCPs in the asymmetric unit of the pentasymmetron (Fig. 3C) to maintain the spiral assembly pathway of the capsid as proposed in other giant viruses (6).

### Three types of spikes

As previously reported (4), medusavirus capsids are decorated with three types of spikes: regular spike, long spike, and wide spike (Fig. 1D). Here, we found that these spikes interacted with MCP trimers in different ways (Fig. 4). The regular spike and the long spike interacted with MCP trimers in a similar manner, with the root of the spike sandwiched between loops 1 and 2 of MCP (black and white arrows in Fig. 4C). As a result, the interaction occurred over a wider range of areas. In contrast, the wide spike interacted with the center of the MCP trimer, and its interactions were formed only with loops near the center, where in addition to two portions of DE2 loop (white arrows in the middle panels of Fig. 4C) in loops 1 and 2, respectively, as described above, FG1 loop (white asterisk in the middle panels of Fig. 4C) of JR1 was located. Interestingly, the FG1 loop in MCP of medusavirus had three α-helices, whereas the FG1 loops of PBCV-1, ASFV, and SGIV only have two. The third α-helix located between JR1 and JR2 and near the center of the MCP trimer uniquely interacted with the wide spike (white asterisk in Fig. 4C) (“wide spike-interacted helix” in Fig. S3), although the individual functions of the different types of spikes are not clear at present.

### Minor capsid proteins around the fivefold vertices

**Fig 5:**
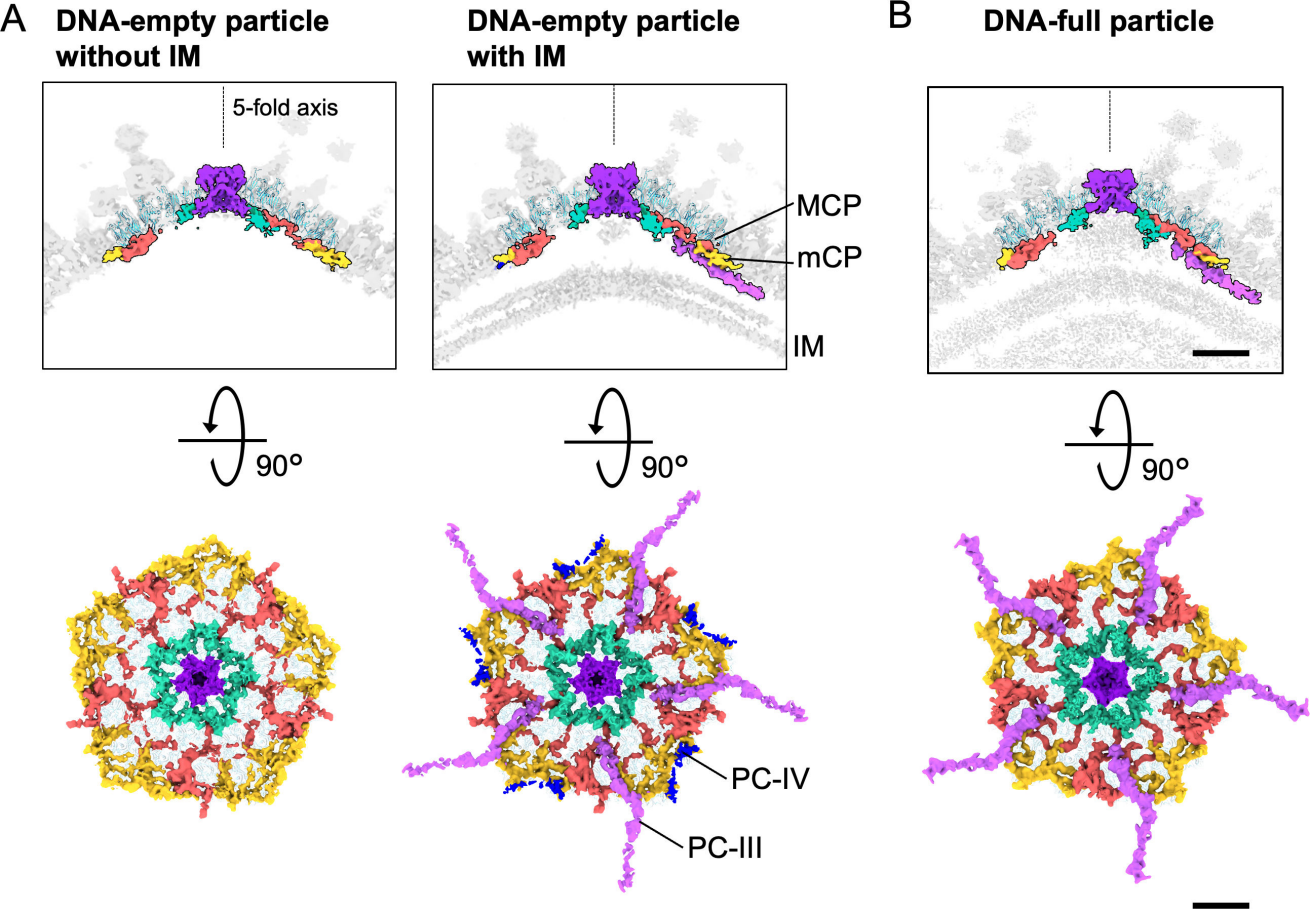
mCP components in the DNA-empty particle without IM, the DNA-empty particle with IM, and the DNA-full particle. (**A and B**) Vertical slices of fivefold maps colored corresponding to Fig. 2 (top panel), and the mCP components viewed from inside the capsid (bottom panel). Maps show blocks of the DNA-empty particles with and without IM (**A**) and the DNA-full particle (**B**). Scale bar = 100 Å.

At the fivefold vertices, the structure of the mCPs changed dramatically depending on the type of particle and the presence or absence of an IM. In DNA-empty particles, there were two types of vertices in the same capsid. The first is the presence of an IM below the vertex (with IM in Fig. 5A), and the second is the absence of an IM (without IM in Fig. 5A) due to the partially open structure. In DNA-empty vertices with IM (right panel in Fig. 5A), PC-IIIs and PC-IVs were located between the mCPs and IM, whereas in DNA-empty vertices without IM (left panel in Fig. 5A), these are not visible. This suggests that PC-IIIs and PC-IVs are required to maintain the IM under mCPs, although a direct interaction with the IM is not clear. Interestingly, in the DNA-full vertices, PC-IVs disappear and only PC-IIIs remain. It is not clear whether these components are lost or have become disordered. However, this suggests that PC-IVs are required to maintain the IM at early stages, but not after the IM has been filled with viral DNA (Fig. 5A and B).

### Penton protein in DNA-empty and DNA-full particles

**Fig 6:**
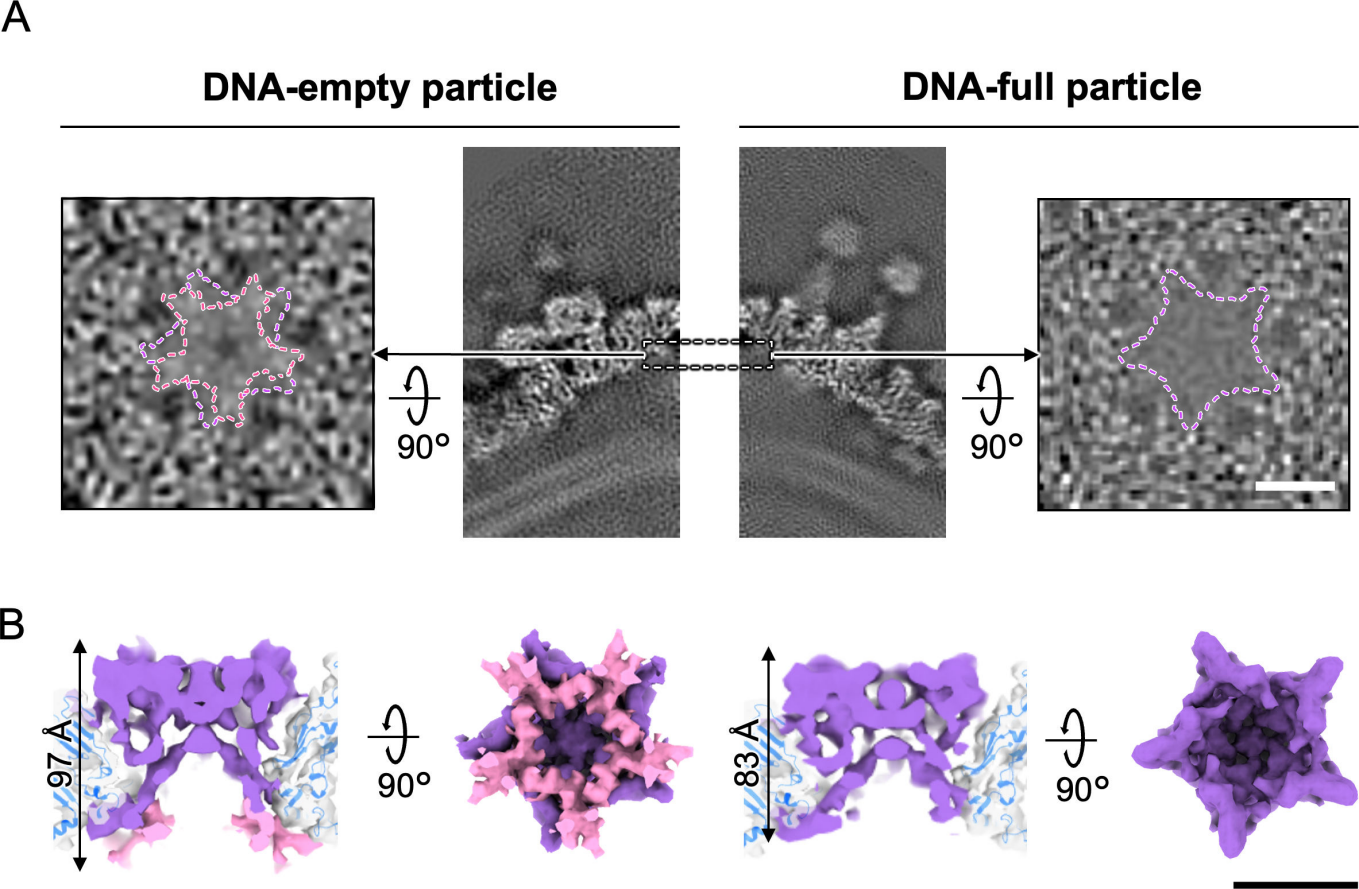
Structural changes of the penton proteins between DNA-empty and DNA-full particles. (**A**) Horizontal and vertical slices around the penton of the DNA-empty (left panels) and DNA-full (right panels) particles as viewed from inside the capsid. The vertical slice of DNA-full particle was clipped and enlarged from Fig. 1A. (**B**) Penton structures of the DNA-empty particle (left panel) and the DNA-full particle (right panel). Differences are highlighted in pink. Scale bars = 50 Å.

Structural changes depending on particle type were also observed in the pentons on the fivefold vertices (Fig. 6). The penton density in DNA-full particles has been shortened by 14 Å compared to DNA-empty particles, indicating that the lower part of the penton protein (pink in Fig. 6B) was truncated or disordered. This may be due to contraction of the IM upon DNA packaging.

### Hook components along twofold axis

**Fig 7:**
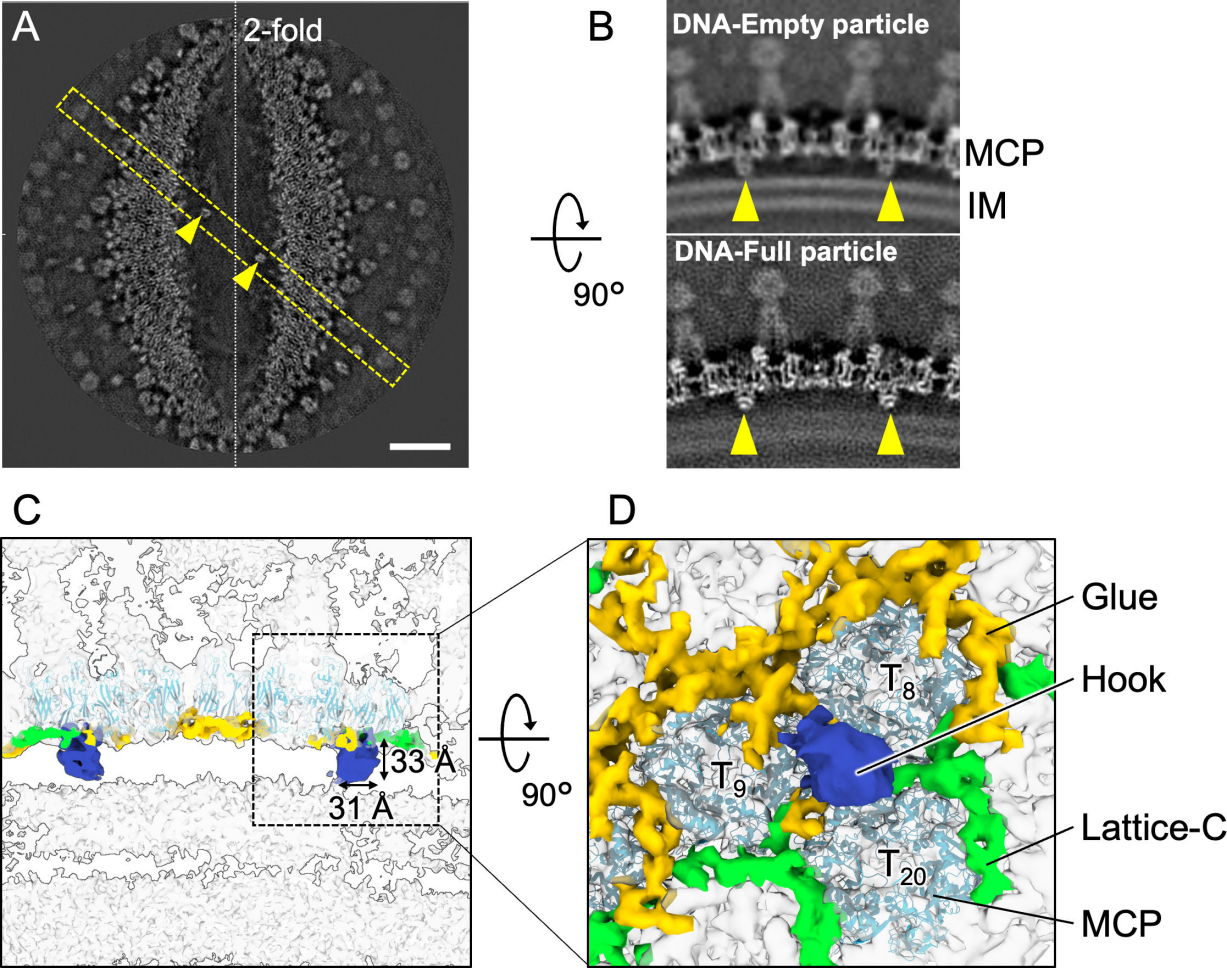
Hook component connects the capsid and the IM along the twofold axis. (**A**) A horizontal slice of the twofold cryo-EM map between the capsid and IM. Scale bar = 200 Å. (**B**) Vertical slices of the DNA-empty and DNA-full particle maps at the yellow dashed box in (A). Hook components are indicated with arrowheads. (**C**) A vertical slice of the cryo-EM map of the Hook components along the twofold axis. The Hook components are in blue. The size of the component is indicated. (**D**) A focused view of the dashed box in C from inside the particle, showing the location of the Hook components within the trisymmetron mCP network numbered and interactions with Lattice-C and Glue components.

The mCP components named “Hook” were symmetrically located along the trisymmetron interface that connected the capsid and the IM (yellow arrows in Fig. 7A through C). The Hook components were bound to the middle of three MCPs, labeled T8, T9, and T20, via the Glue and Lattice-C mCP components (Fig. 7D). These Hook components function to maintain the IM structure in both DNA-empty and DNA-full particles (Fig. 7B).

## DISCUSSION

Here, we showed that the resolution of the medusavirus capsid structure was significantly improved from ~20 Å in our previous study (4) to 7–10 Å using a block-based reconstruction method (10). In this method, localized reconstruction using symmetric blocks, in addition to the Ewald sphere correction, contributed to the resolution improvement. As a result, we were able to identify some structural differences between different types of capsids that represent the virus maturation process. With the use of this block-based reconstruction method, structures of giant virus capsids have been recently reported at near-atomic resolutions of around 3 Å (7–9, 13). For medusavirus capsid, the micrographs were collected with a pixel spacing of 3.03 Å, but the Fourier shell correlation (FSC) curves dropped before reaching the Nyquist frequency (Fig. S1I). This indicates that the capsid proteins do not adopt a constant structure on the capsid beyond this resolution. In particular, the resolution of the outer spikes was significantly reduced at local resolution (Fig. S1). Applying a local mask to remove the spikes improves the resolution of the capsid part somewhat, but it is still far from near-atomic resolution. At these intermediate resolutions, it was not possible to determine whether these structural differences were due to flexibility or lability of the capsid proteins. However, the dynamics of the capsid proteins during the maturation process were suggested to play an important role in the medusavirus life cycle (4).

Because the intermediate resolution achieved here does not allow for the model building of individual mCP structures from the genome, we divided the mCP complexes into components based on their characteristics. However, these classified mCP components are useful for describing the structure and the dynamics of medusavirus capsids. In the future, further improvements in resolution, perhaps by novel methods, will allow the identification of individual mCPs within each component.

As mentioned above, local resolution calculations showed that the regions of the outer spikes were worse than those of the MCPs and mCPs within the medusavirus capsid (Fig. S1). Although we cannot determine whether this is due to structural flexibility, pseudo-symmetry, or lability of individual spikes, the different densities of these spikes represent different properties on the capsid, regardless of particle type. For example, in addition to all long spikes, the wide spikes on three MCP trimers P3, P6, and T3 showed strong density (Fig. S4A). There, the wide spikes on the MCP trimers P3, P6, and T3 showed interactions between them at some points (black and white stars in Fig. S4A and C). Moreover, the long spikes on the MCP trimer T2 also showed interactions with the regular spike on the MCP trimer T14 (black asterisk in Fig. S4A and C). These intermolecular interactions contribute to the stability of these spikes and give rise to their strong intensity in the cryo-EM maps.

On the other hand, no such interactions were observed between the weak wide spikes on the MCP trimers P5 and T1 (Fig. S4A and C). For detailed comparison, we defined a central axis on each MCP trimer attached to a wide spike and measured the tilt angle between them (Fig. S4B). Under the dense wide spikes, the axes of each MCP trimer were not parallel but tilted inward at angles of 13.2° between P3 and P6 and 3.0° between P6 and T3. In contrast, under the weak wide spikes, the axes of each MCP trimer were tilted inward at angles of 17.9° between P3 and P5 and 6.2° between P5 and T1 (Fig. S4B). When the typical cryo-EM map of a wide spike was fitted to the respective positions of the weak wide spikes on the MCP trimers, labeled P5 and T1, an overlap occurred (red area in Fig. S4B). This indicates that the angles between the MCP trimers labeled P5 and T1 are not suitable for forming molecular interactions between the wide spikes. Therefore, the weak density of these wide spikes on P5 and T1 may indicate that these spikes are partially occupied or dislodged during the sample preparation process (Fig. S4B).

We confirmed that the MCP trimer located at the corner of the asymmetric unit of the pentasymmetron was rotated by 60° (Fig. 3C), like other giant viruses previously reported (6). Furthermore, we investigated the tilt angle of each MCP trimer in the asymmetric unit of the pentasymmetron, in comparison with MCP trimer P1 (Fig. S5). The results showed that the tilt angles of each MCP trimer are quite similar to those of other giant viruses that do not have spikes, strongly suggesting that MCP trimers are first assembled to build the capsid, before spikes are decorated on the MCP array. Although wide and long spikes appeared to decorate more tilted MCP trimers compared to those of regular spikes (Fig. S5), some of these spikes were eventually stabilized by interactions with neighboring spikes. In the case of wide spikes, the unique third α-helix in the FG1 loop of the medusavirus MCP may enable spikes to be connected even with a large tilt of the MCP trimer (Fig. 4; Fig. S3).

Previous studies have reported that the open structures in the IM are finally closed during the morphological transition from DNA-empty to DNA-full particles, resulting in a particle size reduction of approximately 1 nm (4). In this study, we observed structural changes in which PC-IIIs, PC-IVs, and pentons that interact with the IM were deformed or partially lost among them (Fig. 5 and 6). Meanwhile, despite the dramatic changes in the IM during maturation, the distance between the capsid and the IM via the Hook component remained constant (Fig. 7B). This suggests that the Hook component plays an important role in maintaining the IM morphology throughout the maturation process.

Two membrane proteins are predicted in medusavirus (encoded by the orf226 and orf329 genes) (1). The membrane protein encoded by the orf226 gene shows homology to membrane proteins of the *Marseilleviridae*. Meanwhile, the myristoylated membrane protein encoded by the orf329 gene shows homology to VP88, an anchor protein of SGIV (8), by PSI-BLAST search (18). VP88 is an mCP that contacts the inner membrane at the trisymmetron-pentasymmetron interface of SGIV. Compared with VP88, the myristoylated membrane protein of medusavirus is predicted as a strong candidate for PC-III, PC-IV, or Hook. Higher resolution of these densities will be required to elucidate homologous mCPs in the future.

The MCP of medusavirus shows high homology with other giant viruses as shown in PBCV-1 and SGIV (Fig. S4A), and the orientation pattern of the MCP trimer on the capsid surface is also like other giant viruses (Fig. 3C). However, except for VP88 of SGIV mentioned above, no mCP homologues have been identified in medusavirus genomes. Tape-measure protein (TMP), a previously commonly identified mCP in several icosahedral giant viruses such as PBCV-1 and ASFV (7 and 13), and in bacteriophages of PRD1 and Bam35 (19, 20), has been proposed to determine the size of the capsid by connecting the fivefold vertices (21). Unfortunately, the current resolution is insufficient to resolve this, and no corresponding density has been found within medusavirus capsids at this time and no homologue identified. Although PC-III is in a position where TMP passes through, the thick density does not fit if TMP is a single peptide chain (7). Similarly, structures equivalent to the TMP have not yet been identified for Chilo iridescent virus (CIV) (22), tokyovirus (12), or melbournevirus (11). These resolutions are also insufficient to directly resolve this. Therefore, we cannot exclude the possibility that these giant viruses, including medusavirus, have alternative mechanisms for determining the size of their large capsids. The elucidation of high-resolution structures of these virus capsids is awaited.

In this study, we investigated the dynamic structural changes of the mCP components of different types of medusavirus particles to reveal the structural changes of the capsid that appear at each maturation stage (4). Some mCP components undergo structurally changed as maturation proceeded, suggesting that these components exist transiently during maturation and are lost or disordered after maturation. This is because medusaviruses are released as immature particles in addition to mature particles. This is the first time that we have been able to report these transient mCP components in a giant virus. The discovery of these transient components is important for elucidating the entire particle maturation process of giant viruses. Medusaviruses are unique in that we can examine immature particles in addition to mature virus particles. Further investigation of the structure of medusavirus particles in the future should shed light on the mechanisms by which giant viruses are formed.

## MATERIALS AND METHODS

### Medusavirus growth and purification

Medusavirus was propagated and purified by infection of *A. castellanii* strain Neff as previously described (4). Briefly, amoeba cells were cultured in flasks containing 100 mL of peptone-yeast-glucose (PYG) medium at 26°C, and medusavirus was harvested 3 days after infection. Amoeba cells and cell debris were removed by centrifugation at 800 ×  g for 5 min at 24°C, and the supernatant was centrifuged at 8,000 ×  g for 35 min at 4°C to pellet the viral particles. The virus particles were suspended in phosphate-buffered saline (PBS) and purified using a 0.45 µm filter (Millex-AA; Merck Millipore, Darmstadt, Germany). The filtered viral particles were centrifuged at 8,000 ×  g for 35 min at 4°C, and then resuspended in PBS. This process was repeated multiple times to obtain sufficient amounts of medusavirus particles.

### Cryo-EM data collection and processing

The data collection and image processing procedures are described previously (4). In brief, 2.5 mL of purified medusavirus particle suspension was applied to glow-discharged Quantifoil grid (R1.2/1.3 Mo; Quantifoil Micro Tools GmbH, Germany). The grids were blotted with filter paper (blotting time, 7 s; blotting force, 10; defined in Vitrobot settings) and plunge-frozen at 4°C with 95% humidity using a Vitrobot Mark IV (Thermo Fisher Scientific, USA). The frozen grids were imaged at 300 kV using a Titan Krios G2 microscope equipped with a Falcon III detector. The data set was recorded at a nominal magnification of ×22,500, corresponding to 3.03 Å per pixel on the specimen. A low-dose technique (exposure of 10 electrons per Å^2^ for 1 s) was used, and the total number of electrons accumulated on the specimen was ~30 electrons per Å^2^ with an exposure time of 3 s. Individual micrograph movies were subjected to frame-by-frame drift correction with MotionCor2 (23), and the contrast transfer function parameters were estimated by CTFFIND4 (24). A total of 4,625 DNA-empty and 7,038 DNA-full particles were then manually selected and extracted from 2,084 motion-corrected images using RELION3.0 software (25). A total of 4,551 DNA-empty particles and 6,981 DNA-full particles were selected from the two-dimensional classification and used for 3D reconstruction imposing icosahedral symmetry. The overall capsid resolution of DNA-empty and DNA-full particles was estimated to be 21.5 Å and 19.5 Å using the 0.143 gold standard FSC criterion (26).

The reconstructed 3D maps were further improved using a block-based reconstruction strategy (10) that compensates for the defocus gradient across, and particle flexibility of, large particles. The image processing workflow for block-based reconstruction is summarized in Fig. S1. First, symmetry expansion was performed using 2 × downsampled images. Then, three masks focused individually on the fivefold, threefold, and twofold axes were generated with UCSF Chimera (27) before particle subtraction was used to calculate the shifts to center the in-focus region within the box. After shift calculation, particle subtraction was cancelled. Using these RELION-calculated shifts, we extracted the fivefold, threefold, and twofold axes with boxes of 256, 400, and 512 pixels, respectively. All block reconstructions were performed with imposing C1 symmetry, and the fivefold block reconstructions were performed with C5 symmetry after removing symmetry-expanded symmetry mates. The “relion_reconstruct” module was used for each extracted particle set to generate references for refinement and to ensure that the positioning was correct. For each segment, 3D refinement was performed using non-downsampled particle images. Defocus refinement was performed once for each segment, and the half-map was reconstructed using “relion_reconstruct” with the refined particle parameters for defocus. Reconstruction details are summarized in Table 2.

**TABLE 2:** Reconstruction details

	Fivefold block			Threefold block		Twofold block	
Particle types	DNA-Empty		DNA-Full	DNA-Empty	DNA-Full	DNA-Empty	DNA-Full
	With IM	Without IM					
Particles	19,028	21,900	67,456	127,482	148,795	208,834	189,286
Symmetry	C5	C5	C5	C1	C1	C1	C1
Resolution	8.62	8.81	7.32	9.93	8.54	9.88	8.92

Cryo-EM maps of the MCP was interpreted by fitting the molecular model generated by AlphaFold2 (14). The fitted model was manually corrected by COOT (15) and refined by PHENIX (17) (Table 3).

**TABLE 3:** Refinement and validation statistics

Map	Threefold block (DNA-full)
Initial model	AlphaFold2 generated model
R.m.s. deviations	
Bond lengths (Å)	0.004
Bond angles (°)	0.983
Validation	
MolProbity score	2.41
Clashscore	25.60
Ramachandran plot (%)	
Favored	91.12
Allowed	8.57
Outliers	0.31

## Data Availability

The cryo-EM maps have been deposited in the EMDB with the accession codes EMD-39292 (5-fold block of DNA-full particles), EMD-39293 (5-fold block of DNA-empty particles with IM), EMD-39294 (5-fold block of the DNA-empty particle without IM), EMD-39295 (3-fold block of DNA-full particles), EMD-39296 (3-fold block of DNA-empty particles), EMD-39297 (2-fold block of DNA-full particles), and EMD-39298 (2-fold block of DNA-empty particles).

## SUPPLEMENTAL MATERIAL

Supplemental material jvi.00436-24-s0001.pdf Fig. S1 to S5.

ASM does not own the copyrights to Supplemental Material that may be linked to, or accessed through, an article. The authors have granted ASM a non-exclusive, world-wide license to publish the Supplemental Material files. Please contact the corresponding author directly for reuse.
