# Supplementary material for: Subnanometer structure of medusavirus capsid during maturation using cryo-electron microscopy
Source: J Virol. 2024 Aug 28;98(9):e00436-24. doi: 10.1128/jvi.00436-24 (PMC11406985; doi:10.1128/jvi.00436-24)
Supplement: Supplemental material — Fig. S1 to S5. [file jvi.00436-24-s0001.pdf]

Supplementary information

Subnanometer structure of medusavirus capsid during maturation using cryo-electron microscopy

Ryoto Watanabe[1,2,3], Chihong Song[1,2,3], Masaharu Takemura[4], Kazuyoshi Murata[1,2,3*]

[1] Department of Physiological Sciences, School of Life Science, The Graduate University for Advanced Studies (SOKENDAI), Okazaki, Aichi, 444-8585, Japan

[2] Exploratory Research Center on Life and Living Systems (ExCELLS), National Institutes of Natural Sciences, Okazaki, Aichi, 444-8585, Japan

[3] National Institute for Physiological Sciences, National Institutes of Natural Sciences, Okazaki, Aichi, 444-8585, Japan

[4] Institute of Arts and Sciences, Tokyo University of Science, Shinjuku, Tokyo, 162-8601, Japan

* Corresponding author and Lead contact: Kazuyoshi Murata (kazum@nips.ac.jp)

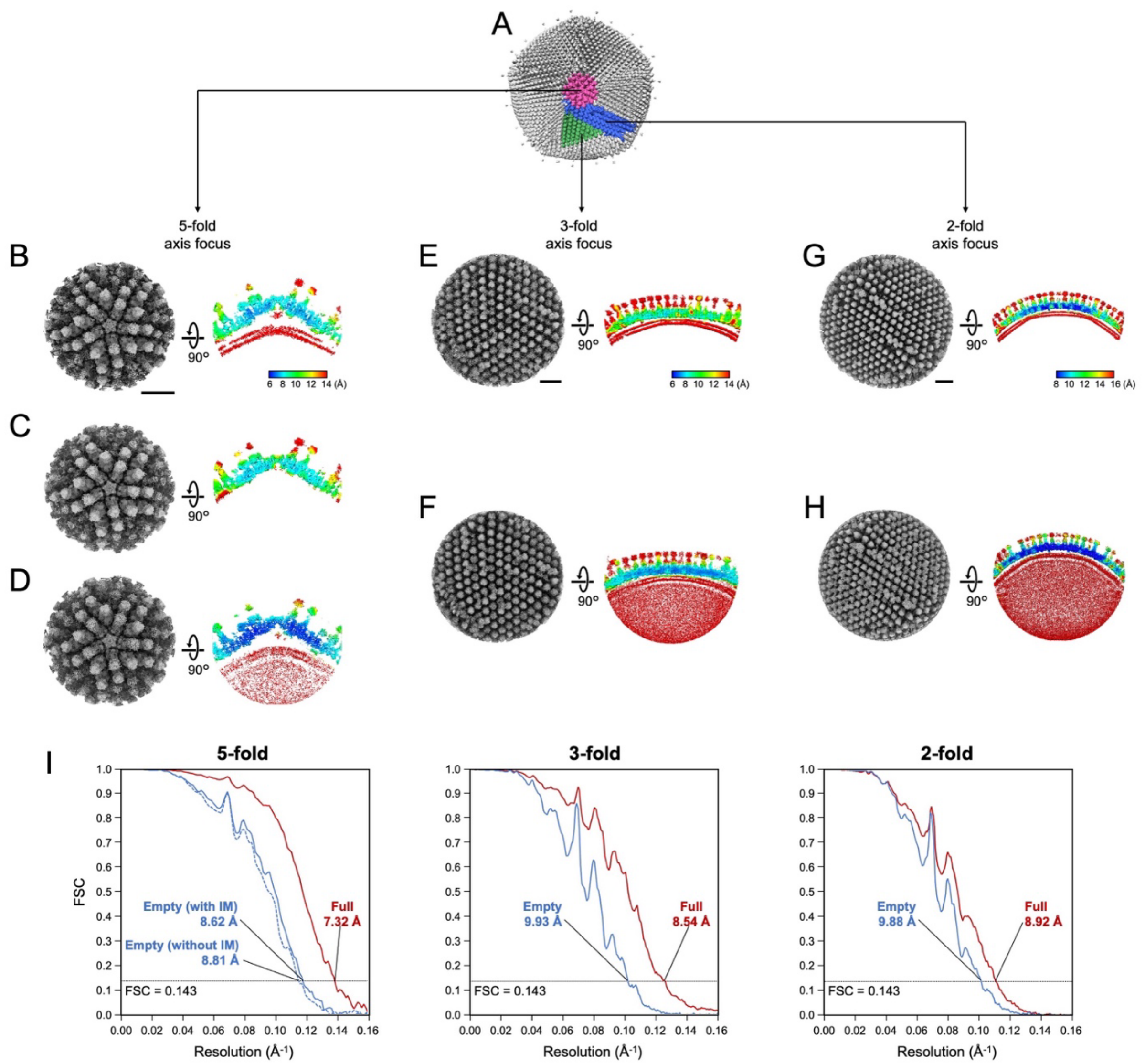

Fig. S1 Single particle image processing flowchart for the block-based reconstruction. (A) Blocks centered on the 5, 3, and 2-fold axes of the medusavirus capsid are colored pink, green, and blue, respectively. (B, C and D) Cryo-EM maps of the 5-fold block (left panel), and the vertical slices of the maps along the 5-fold axis colored by local resolution (right panel). The maps show the DNA-empty particle with IM (B), the DNA-empty particle without IM (C), and the DNA-full particle (D). (E and F) Cryo-EM maps of the 3-fold block (left panel), and the vertical slices of the maps along the 3-fold axis colored by local resolution (right panel). The

maps show the DNA-empty particle (E), and the DNA-full particle (F). (G and H) Cryo-EM maps of the 2-fold block (left panel), and the vertical slices of the maps along the 2-fold axis colored by local resolution (right panel). The maps show DNA-empty particle (G), and DNA-full particle (H). Scale bars = 200 Å. The local resolution values are color-coded, respectively. (I) Gold standard FSC curves of 5-fold, 3-fold, and 2-fold blocks, respectively. Curves of DNA-full and DNA-empty particles with and without IM are included in different colors.

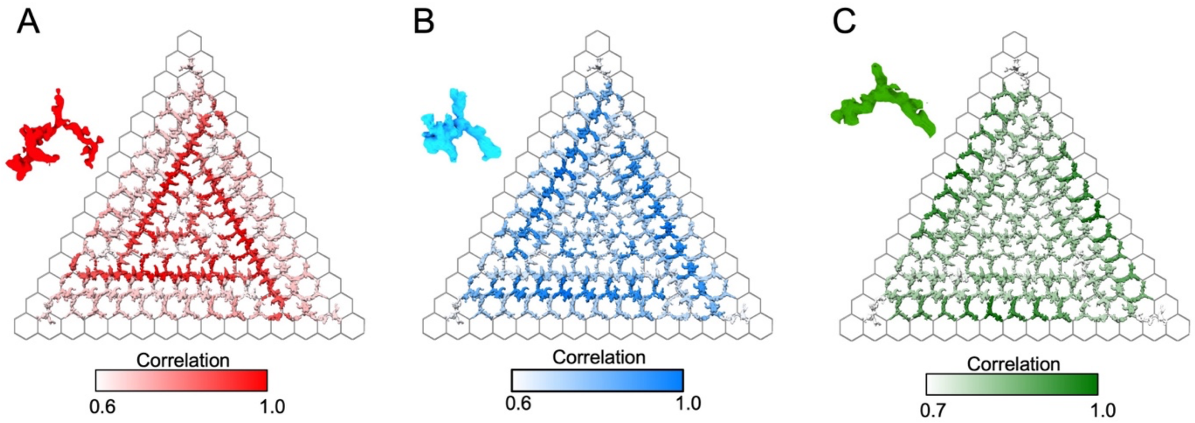

Fig. S2 Three lattice units and their correlation in the trisymmetron mCP network. The correlations between three lattice units (Lattice-A, B, and C) (red, light blue, and green) in the trisymmetron mCP network were calculated, and the correlation values are color-coded.

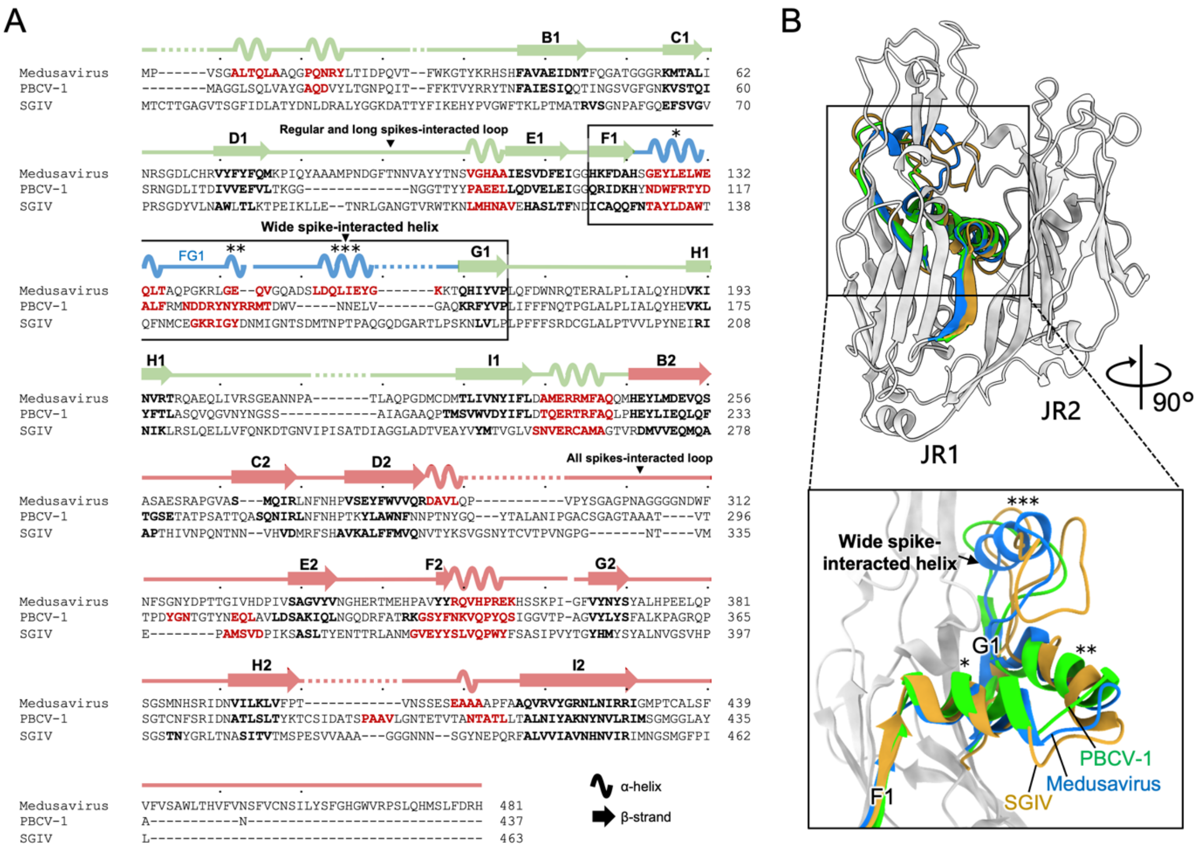

Fig. S3 Sequence alignment of MCP and comparison of the helix in FG1 loop. (A) Multiple alignment of MCP sequences among MedV, PBCV-1, and SGIV. These MCPs are commonly composed of "double jelly roll" motif, as shown in JR1 (lightgreen) and JR2 (Salmon). (B) Overlay of MCP ribbon diagrams of medusavirus (sky blue), PBCV-1 (light green), and SGIV (orange). Focused views of the dashed box is shown to compare FG1 loops between these viruses. The third α-helix (***) in FG1 loop was uniquely observed in the medusavirus MCP.

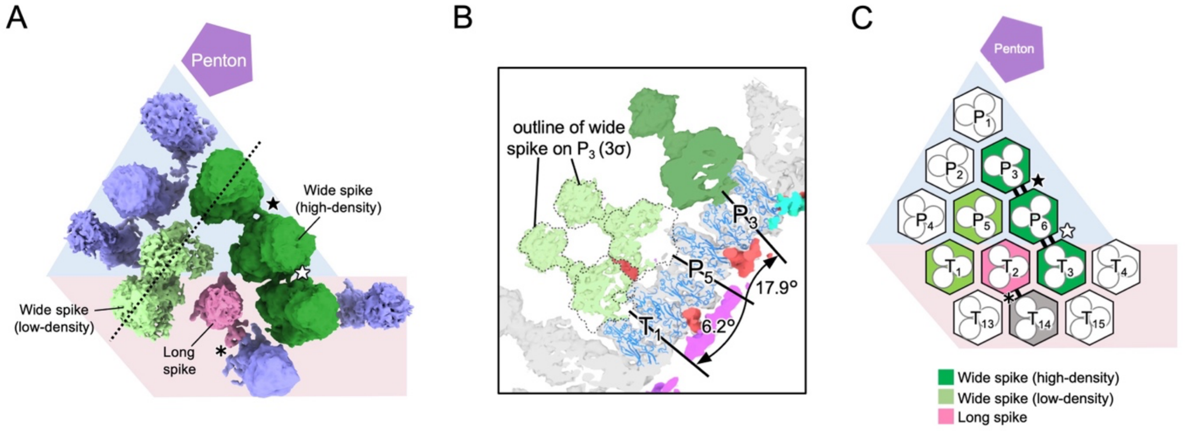

Fig. S4 A cryo-EM map showing the three types of spikes and their interactions in the pentasymmetron asymmetric unit and the adjacent trisymmetron. (A) A cryo-EM map of the three types of spikes in the pentasymmetron asymmetric unit and the adjacent trisymmetron are indicated. Each spike type is color-coded corresponding to Fig. 4A. Interactions between spikes are marked with black stars, white stars, and asterisks. Contour level = 1σ. (B) Vertical slice of the cryo-EM map at the dashed line in A. Radial angles between neighboring spikes are indicated. (C) Schematic diagram shows the three types of spikes and their interactions in the pentasymmetron asymmetric unit and the adjacent trisymmetron.

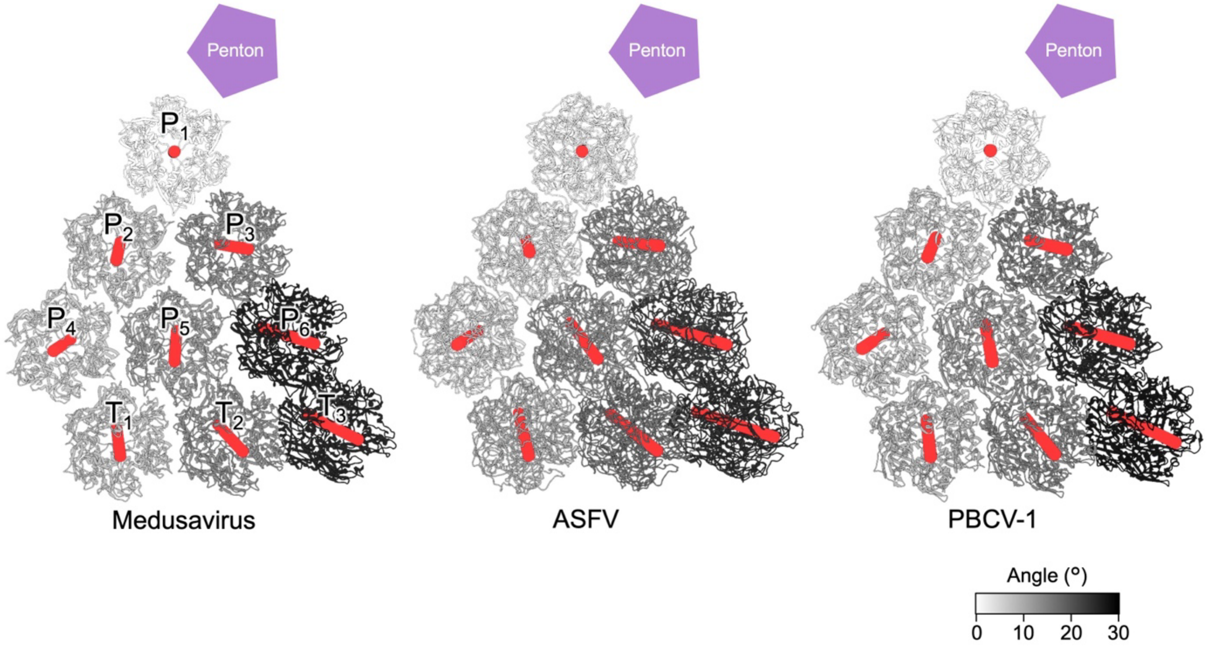

Fig. S5 Relative tilted angles of each MCP trimer compared to the MCP trimer (P1) adjacent to the penton of various viruses. MCP trimers (P1 to P6, and T1 toT3) are color-coded according to the tilted angles indicated. The tilt directions are indicated with bars.
